# Comparative Transcriptome Analyses Uncover Key Candidate Genes Mediating Flight Capacity in *Bactrocera dorsalis* (Hendel) and *Bactrocera correcta* (Bezzi) (Diptera: Tephritidae)

**DOI:** 10.3390/ijms19020396

**Published:** 2018-01-30

**Authors:** Shaokun Guo, Zihua Zhao, Lijun Liu, Zhihong Li, Jie Shen

**Affiliations:** Key Laboratory of Ministry of Agriculture for Monitoring and Green Management of Crop Pests, Department of Entomology, College of Plant Protection, China Agricultural University, Beijing 100193, China; mscgsk@163.com (S.G.); zhzhao@cau.edu.cn (Z.Z.); ljliu@cau.edu.cn (L.L.)

**Keywords:** *Bactrocera dorsalis* Hendel, *Bactrocera correcta* Bezzi, flight capacity, transcriptome, RNA interference, *EGFR*, energy metabolism, juvenile hormone

## Abstract

Flight capacity is important for invasive pests during entry, establishment and spreading. Both *Bactrocera* *dorsalis* Hendel and *Bactrocera* *correcta* Bezzi are invasive fruit flies but their flight capacities differ. Here, a tethered flight mill test demonstrated that *B. dorsalis* exhibits a greater flight capacity than *B. correcta*. RNA-Seq was used to determine the transcriptomic differences associated with the flight capacity of two *Bactrocera* species. Transcriptome data showed that 6392 unigenes were differentially expressed between the two species in the larval stage, whereas in the adult stage, 4104 differentially expressed genes (DEGs) were identified in females, and 3445 DEGs were observed in males. The flight capacity appeared to be correlated with changes in the transcriptional levels of genes involved in wing formation, flight muscle structure, energy metabolism, and hormonal control. Using RNA interference (RNAi) to verify the function of one DEG, the epidermal growth factor receptor (*EGFR*), we confirmed the role of this gene in regulating wing development, and thereby flight capacity, in both species. This work reveals the flight mechanism of fruit flies and provides insight into fundamental transcriptomics for further studies on the flight performance of insects.

## 1. Introduction

**Figure 1:**
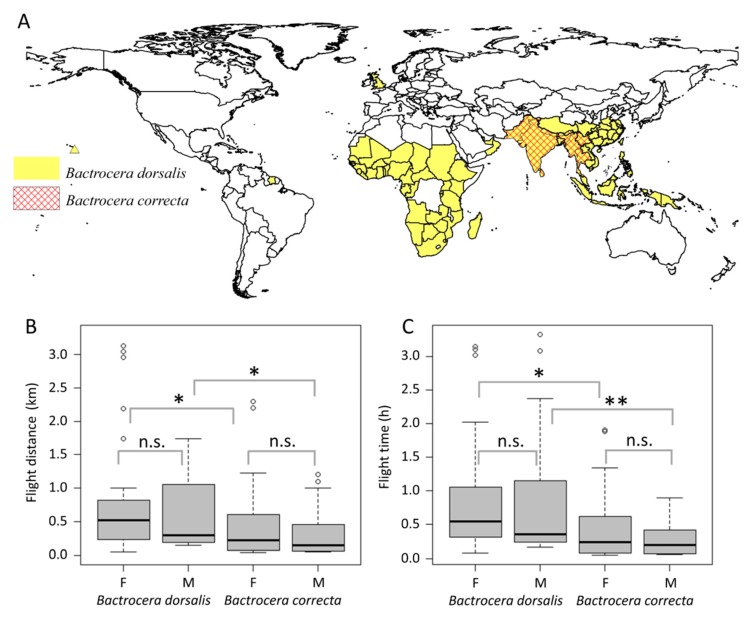
The geographical distribution and flight capacity of *B. dorsalis* and *B. correcta.* (**A**) The world map shows countries where the indicated species has been reported. The yellow region indicates the distribution of *B. dorsalis*, and the red quilting indicates the distribution of *B. correcta.* The map was created using ArcGIS 10.2 software (ESRI Inc., Redlands, CA, USA). Available online: http://www.esri.com/arcgis/about-arcgis; (**B**) The comparison of flight distances between *B. dorsalis* and *B. correcta*; (**C**) The comparison of flight time between *B. dorsalis* and *B. correcta*. The circles show the outliers, the bold black line shows the median, the top edge of the bar shows the sample maximum and the lower edge of the bar shows the sample minimum. (F: female; M: male; n.s.: not significant; * *p* < 0.05, ** *p* < 0.01, according to the Student’s *t* test).

The attention given to invasive species continues to grow because these species pose a major threat to biodiversity and agricultural production [1,2]. *Bactrocera dorsalis* Hendel and *Bactrocera correcta* Bezzi are reported as invasive fruit flies and are treated as quarantine pests in many countries [3,4,5,6]. *B. dorsalis* and *B. correcta* have many similarities in terms of habits and hosts [6,7]. However, *B. dorsalis* has spread to nearly 70 countries [7] while *B. correcta* is limited to South and Southeast Asian countries [6] (Figure 1A). *B. dorsalis* has been found in 17 provinces in China, with a trend toward northern expansion [8,9]. In contrast, *B. correcta* is limited in Yunnan and Sichuan provinces [10] (Figure 1A). We suspect that there are differences in the two species’ flight capacity.

A tethered flight mill system is commonly used to test insect flight behaviour. In such studies, a number of insects, including the oriental migratory locust (*Locusta migratoria manilensis* Meyen), the Asian gypsy moth (*Lymantria dispar* Linnaeus), the rice leaf folder (*Cnaphalocrocis medinalis* Guenée), and the white-backed plant hopper (*Sogatella furcifera*), have been investigated regarding their flight capacity [11,12,13,14]. Several invasive pests such as potato beetles (*Leptinotarsa decemlineata* Say) and *B. dorsalis* have also been examined using the tethered flight mill system [15,16,17,18]*. B. dorsalis* has a strong natural spreading capacity [19,20], with a maximum flight distance recorded at 65 km [21]. Chen et al. have also found that flight capacity becomes stronger after sexual maturity [18]. However, there are few studies that focus on the flight capacity of *B. correcta*, and a limited number of studies are able to reveal the mechanism underlying the flight capacity.

Transcriptome sequencing has been used to examine the genes involved in insecticide resistance, reproduction and development, and the detoxification mechanisms of *B. dorsalis* [22,23,24]. However, there is little available information about the transcriptional changes related to flight in these two species. RNA interference (RNAi) refers to the highly efficient and specific degradation of homologous mRNA induced by double-stranded RNA (dsRNA). RNAi technology can specifically remove or decrease the expression of specific genes, and this technology has, therefore, been widely used to explore gene functions [25]. Injection-mediated RNAi can effectively knock down various target genes in *B. dorsalis* [26,27,28], and diet-delivered RNAi has been reported to be an effective method of introducing dsRNA [29,30]. However, the genes related to the flight capacity of these two *Bactrocera* species have not been examined.

In the present study, we began by verifying the flight performance of *B. dorsalis* and *B. correcta* which reveals differences in their flight capacity. To obtain insight into the molecular mechanisms underlying the differences, we performed Illumina RNA sequencing (RNA-seq) to characterize differentially expressed genes (DEGs) for the two *Bactrocera* species. Among the identified DEGs, candidate genes involved in wing formation, flight muscle structure, energy metabolism and hormonal control play important roles in flight capacity. Additionally, we investigated the function of one key gene, the epidermal growth factor receptor (*EGFR*), by using RNAi in *B. dorsalis* and *B. correcta*. Our results provide a valuable resource to further understand the molecular mechanisms of the flight performance in invasive fruit flies.

## 2. Results

### 2.1. Flight Capacity Differs Significantly between Two Bactrocera Species

Adults of a laboratorial population were tested on a flight mill and showed no significant differences between males and females from both *B. dorsalis* and *B. correcta* for flight speed, flight time and flight distance (*B. dorsalis*: T_1, 29_ = 0.317, *p* = 0.754; *B. correcta*: T_1, 29_ = −1.243, *p* = 0.224) (Table S1). There was no significant difference in flight speed between the two species (males: T_1, 29_ = 0.364, *p* = 0.719; females: T_1, 29_ = 0.180, *p* = 0.859) (Table S1). *B. dorsalis* was able to fly significantly longer distances (males: T_1, 29_ = 2.697, *p* = 0.012; females: T_1, 29_ = 2.601, *p* = 0.014) and for longer durations (males: T_1, 29_ = 3.382, *p* = 0.002; females: T_1, 29_ = 3.033, *p* = 0.005) than *B. correcta* (Figure 1B,C). *B. dorsalis* was able to fly 1.153 km (females) or 0.85 km (males), with speeds of 0.28 m/s (females) or 0.29 m/s (males) within 12 h. In comparison, female *B. correcta* flew 0.464 km, with a speed of 0.27 m/s, and males flew 0.311 km, with a speed of 0.28 m/s. 

### 2.2. Qualitative Description for Assembly and Annotation of Transcriptomes

The Q30 percentage of bases was over 80%, indicating that the sequencing was reliable. We obtained 34,230 unigenes from larval transcriptome sequencing and 110,890 unigenes from adults. The length distribution of the unigenes is shown in Figure S1, and the ratio of unigenes with a length greater than 1000 bp was over 15% (Tables S2 and S3). A total of 19,829 unigenes were annotated in the larval transcriptome, from five databases: NR (non-redundant), Swiss-Prot, GO (Gene Ontology Consortium), COG (Cluster of Orthologous Groups of proteins) and KEGG (Kyoto Encyclopedia of Genes and Genomes) (Table S4). For the adult transcriptome assembly, annotation information was obtained for 26,368 (*B. dorsalis*) and 27,939 (*B. correcta*) unigenes from seven public databases including NR, Swiss-Prot, GO, COG, KOG, KEGG and Pfam, and detailed information about the annotations is provided in Tables S5 and S6. The results of PCA (principal component analysis) indicated distinct transcriptional characteristics for *B. dorsalis* and *B. correcta*, despite the gap between larvae and adults (Figure S2A).

### 2.3. DEGs Annotation in GO, COG and KEGG Databases

A total of 4104 unigenes were differentially expressed in females of *B. dorsalis* compared with *B. correcta*, including 2409 up-regulated and 1695 down-regulated genes. There were 3445 DEGs between males of the two species, 1669 of which were up-regulated and 1776 of which were down-regulated. Among them, 2024 DEGs were common in the two comparisons (Figure S2B).

**Figure 2:**
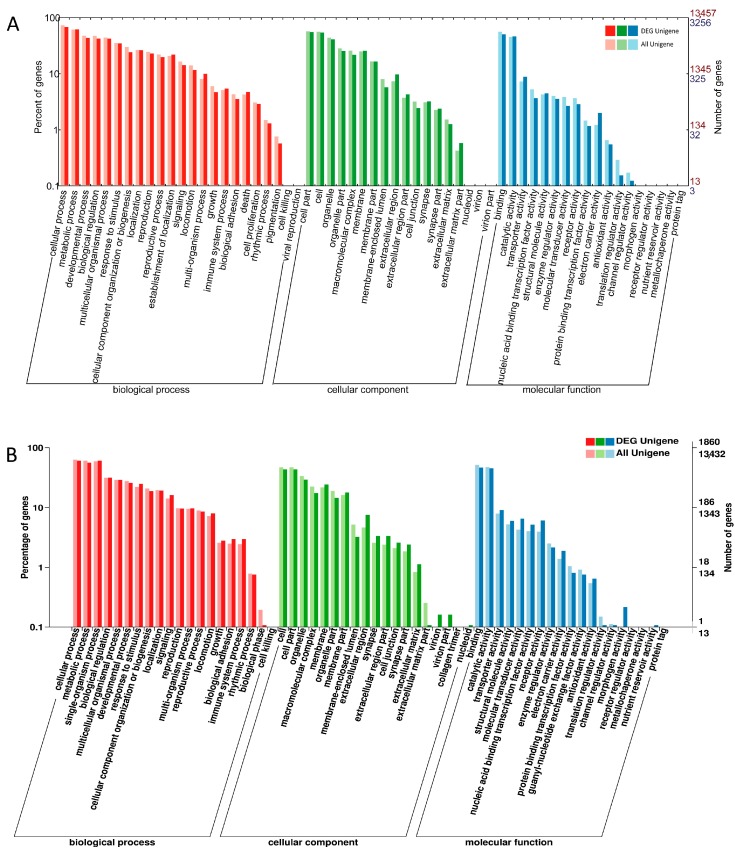

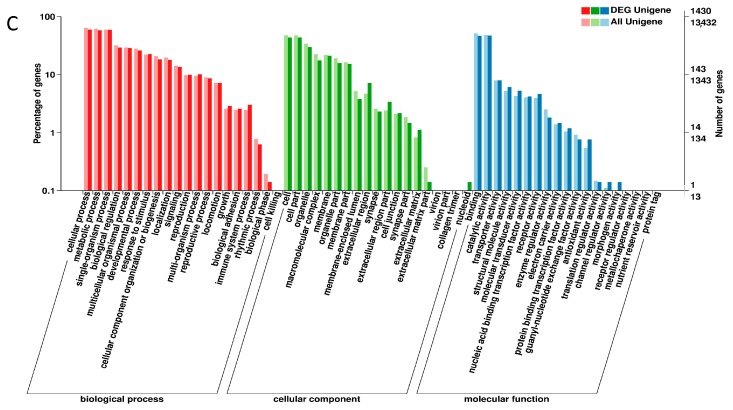
Gene ontology (GO) assignment for the *B. dorsalis* and *B. correcta* unigenes. The abscissa shows the GO content. The ordinate on the left is the percentage of the number of genes, while that on the right is the number of genes. The graphs represent both the number of all unigenes and that of DEGs in (**A**) larvae; (**B**) females and (**C**) males assigned to each GO term.

The GO system classifies genes according to three categories: biological process, cellular component and molecular function. The gene enrichment of GO secondary functions in the background of all unigenes and DEGs is shown in Figure 2. The “extracellular matrix part”, “electron carrier activity” and “translation regulator activity” terms exhibited significantly different proportions in the larvae (Figure 2A). The two species showed significantly different proportions of DEGs in the biological phase, extracellular matrix part and morphogen activity for both females (Figure 2B) and males (Figure 2C).

**Figure 3:**
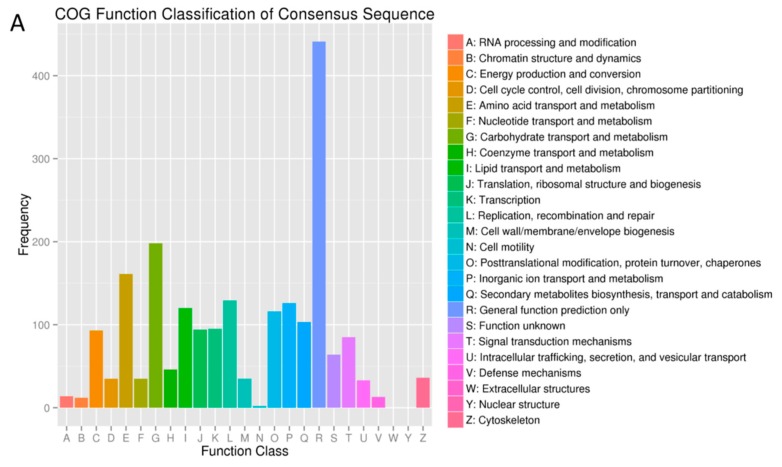

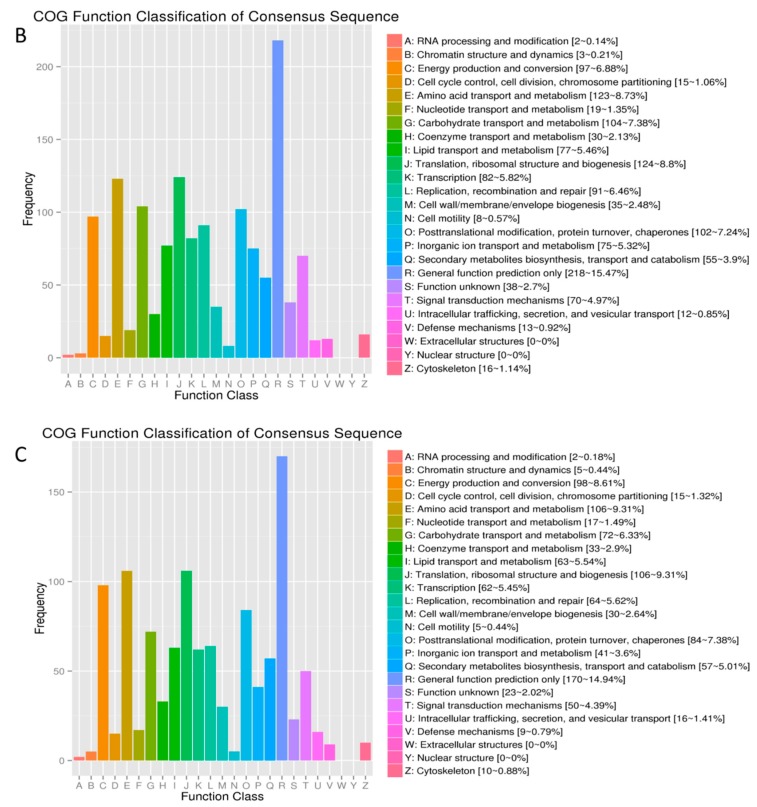
Clusters of orthologous groups (COG) classification of *B. dorsalis* and *B. correcta* unigenes. The abscissa is the COG content of 25 function categories, while the ordinate is the number of genes. The COG classification of DEGs for larvae, females and males was indicated by (**A**), (**B**), and (**C**), respectively.

In the COG database, the largest COG cluster was the general function prediction. Carbohydrate and amino acid transport and metabolism were found to play vital roles in the larvae comparison (Figure 3). Clusters of amino acid and carbohydrate transport and metabolism, energy production, conversion and translation, ribosomal structure and biogenesis were apparent in both female and male comparisons (Figure 3).

**Figure 4:**
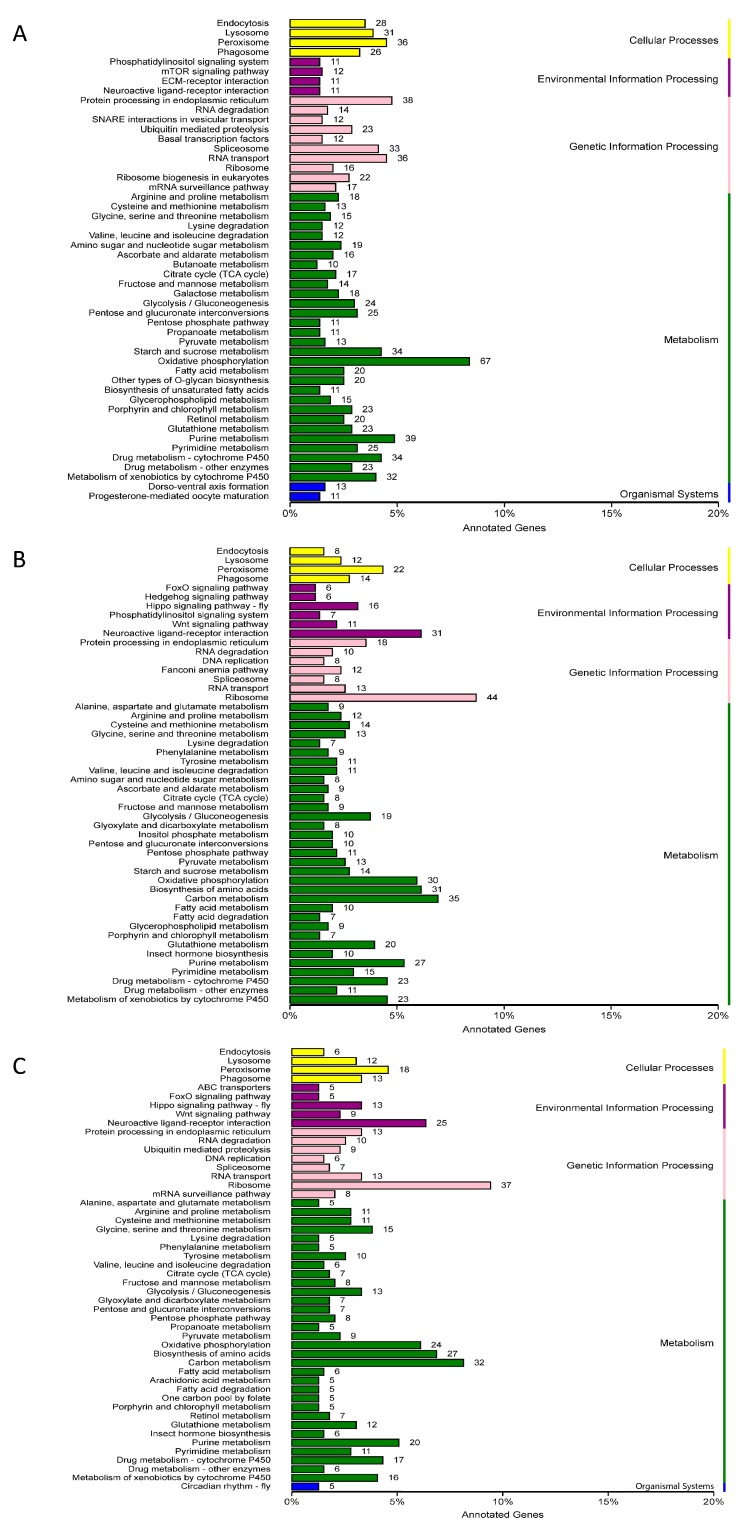
DEGs classified according to the pathway type in KEGG. The abscissa shows the ratio of the number of genes that are annotated to certain pathway and the number of genes that are annotated in total. The ordinate shows the name of the KEGG metabolic pathway. The KEGG classification of DEGs for larvae, females and males were shown by (**A**), (**B**), and (**C**), respectively.

The pathway annotations of the DEGs were classified according to KEGG pathway types. The classification diagram presented in Figure 4 shows that the cluster of oxidative phosphorylation accounted for the largest proportion in larva comparisons and that ribosome accounted for the largest proportion in adult comparisons.

### 2.4. Candidate Genes Involved in Flight Capacity

**Figure 5:**
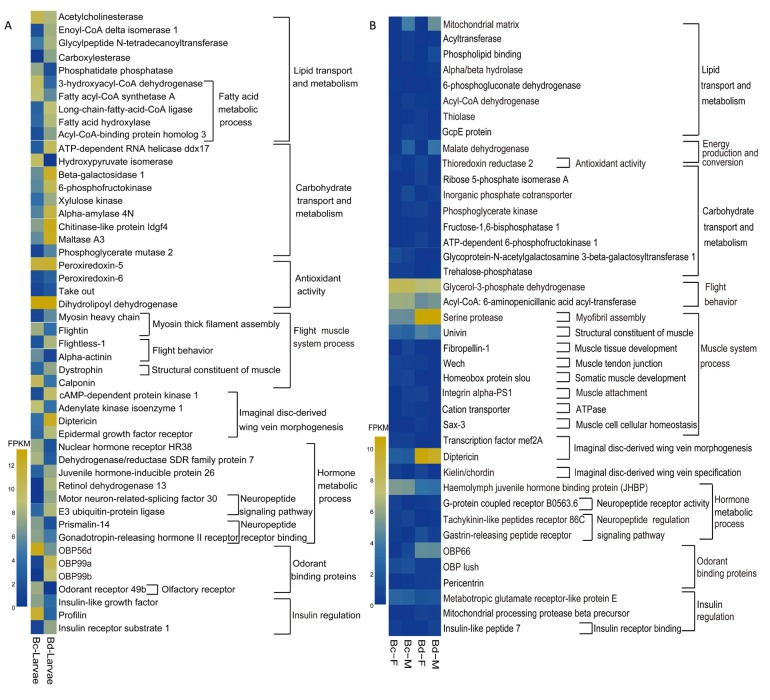

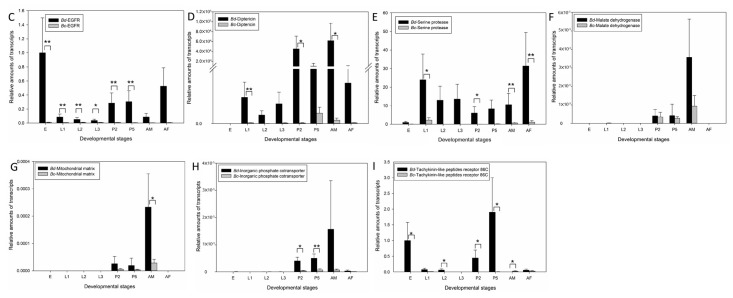
Differential expression and qRT-PCR verification of core genes in *B. dorsalis* and *B. correcta*. Heat maps of absolute expression measured as a normalized FPKM (fragments per kilobase of exon per million reads mapped) value from the perspective of larvae (**A**) and adults (**B**) in two species. The candidate genes tested (**C**–**I**) were selected to cover a range of biological processes contributing to flight. The bars represent the mean ± SD (*n* = 3). (*Bd*: *B. dorsalis*; *Bc*: *B. correcta*; F: female; M: male; E: eggs; L1: 1st instar larvae; L2: 2nd instar larvae; L3: 3rd instar larvae; P2: 2 days pupae after pupation; P5: 5 days pupae after pupation; AM: adult male; AF: adult female; * *p* < 0.05, ** *p* < 0.01, according to the Student’s *t* test).

A heat map was generated to elucidate the differences between the two species (Figure 5). *EGFR* and *diptericin* were annotated to the function of wing vein morphogenesis and were expressed at a higher level in *B. dorsalis* than in *B. correcta* (Figure 5A–D).

A structural constituent gene of the flight muscle, *univin* [31], was largely up-regulated in *B. dorsalis* (Figure 5B). Serine protease SP24D plays an important role in myofibril assembly [32,33], which was expressed at a significantly higher level in *B. dorsalis* than in *B. correcta* (Figure 5B,E). Myofibril filament arrangements directly control muscular contraction [34].

**Figure 6:**
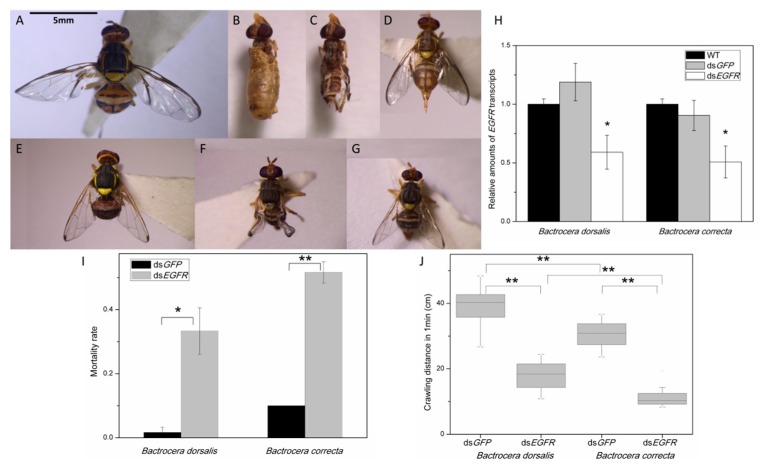
Phenotype analyses and statistics of mortality rates and crawling ability after RNAi. Individuals treated with ds*BdGFP* (**A**) and ds*BcGFP* (**E**) do not differ significantly from the wild types; The non-feathering individuals of *B. dorsalis* (**B**) with fragmentary wings (**C**) in pupae were observed; The deformed phenotypes also included wing-deformed adults (*B. correcta*, **F**) and the smaller-size adults (*B. dorsalis* (**D**) and *B. correcta* (**G**)); The *EGFR* mRNA levels decreased dramatically in ds*EGFR* treated groups compared with control groups, and ds*GFP* treatment did not affect *EGFR* expression (**H**); (* *p* < 0.05 according to One-way ANOVA, Tukey’s multi-comparison *post-hoc* test); Mortality rates of ds*EGFR* and ds*GFP* treated groups were calculated (**I**); and the significant variation in crawling ability between ds*GFP* and ds*EGFR* treated groups were tested (**J**). (* *p* < 0.05, ** *p* < 0.01, according to the Student’s *t* test).

Malate dehydrogenase, a component of lipid particles [35], is an important enzyme involved in carbohydrate aerobic oxidation, and the gene was differentially expressed between *B. dorsalis* and *B. correcta*. Interestingly, the unigenes corresponding to malate dehydrogenase and to the mitochondrial matrix were specifically expressed in males (Figure 5) and the transcription levels were verified by qRT-PCR (Figure 6F,G). The inorganic phosphate cotransporter (IPC) also plays key roles in the synthesis of ATP [36,37], and the unigene was expressed at higher levels in *B. dorsalis* (Figure 6H).

Juvenile hormone (JH) is an important signal involved in insect growth and development. The haemolymph juvenile hormone binding protein (JHBP) protects JH molecules from hydrolysis via esterases present in the insect haemolymph [38]. The unigenes annotated to JHBP was expressed at higher levels in *B. correcta* than in *B. dorsalis* (Figure 5). Tachykinin-86C is a receptor of neuropeptide Tachykinin [39], and the unigenge was expressed at a lower level in *B. dorsalis* (Figure 5B,I). Additionally, insulin regulation-related factors were relatively higher in *B. dorsalis* than in *B. correcta* (Figure 5).

### 2.5. Suppression of EGFR via RNAi Markedly Inhibits Flight

For function verification, we selected *EGFR*, which has an impact on imaginal disc-derived wing vein morphogenesis. The phenotypes resulting from *EGFR* RNAi included the normal phenotype as well as deformed adults and smaller adults. The deformed phenotypes consisted of non-feathering individuals with fragmentary wings in pupae (Figure 6B,C) and deformed wings in adults (Figure 6F). The morphology of the wings of smaller adults was normal, but the overall size of the individuals was smaller than that of the control group (Figure 6D,G). QRT-PCR (quantitative real-time PCR detecting system) showed that *EGFR* mRNA levels decreased dramatically at 48 h after the second feeding of ds*EGFR*, as compared with that in wild-type and ds*GFP*-fed insects. Feeding of ds*GFP* did not affect *EGFR* expression (Figure 6H).

The numbers of smaller flies and flies with deformed wings were used to calculate the deformity rate. The results showed that the mortality rates of ds*BdEGFR*- and ds*BcEGFR*-fed adults were approximately 33.3% and 51.7%, respectively. These values are significantly different from those of the ds*GFP*-treated groups (Figure 6I). The deformity rates in these two groups were 62.5% and 65.5%, respectively, whereas no deformities were observed after ds*GFP* treatment. The surviving insects were completely unable to fly, so we tested their crawling capacity as an indicator of their athletic capacity. The results showed significant differences between the ds*GFP*- and ds*EGFR*-treated groups (Figure 6J).

## 3. Discussion

In order to identify the differences in flight capacity between *B. dorsalis* and *B. correcta*, we compared the flight capacity and transcriptome of the two species. We found that underlying genes from the molecular level can, when coordinated, regulate the flight activity in the two *Bactrocera* species. The results of flight testing proved the differences in flight capacity of *B. dorsalis* and *B. correcta*, suggesting that *B. dorsalis* may exhibit greater endurance than *B. correcta*. The significant over-representation of morphogen activity between two *Bactrocera* adults implies that morphogens might be crucial for the putative flight capacity of the two species. Previous work has revealed that morphogens are associated with the formation of wings and, therefore, may participate in the programming of the flight capacity [40,41]. The functional prediction and classification of unigenes by using the COG database showed that carbohydrate and amino acid transport and metabolism are critical in larval stages, whereas energy production and conversion are important for the development of adults. The most enriched pathway in adults (“ribosome”, serving as the site of translation), played a key role in distinguishing the two species on the basis of biological protein synthesis. The KEGG pathway assignment was instrumental for predicting the functions of genes related to flight, and the obtained results should contribute to further research on relevant biological processes and metabolic pathways.

Adult winged insects can fly, so the adult transcriptome analysis plays an important role in studying flight activity. Furthermore, the development in the larval stage ensures normal adult morphogenesis, and some genes that determine the growth of adults are more active in larval stages, such as wing developmental genes [42,43]. Thus, we conduct a comprehensive analysis of both the larvae and adult transcriptomes. The tubular veins of insect wings are rigid but flexible, thus supporting and reinforcing the wings. Therefore, the wing vein morphogenesis is important for insect flight [44]. *EGFR* is the transmembrane tyrosine kinase receptor for signaling ligands in the TGFα (transforming growth factor α) family [45]. The roles of *EGFR* include developmental patterning [46] and growth regulation in *Drosophila* [47], where it can affect wing disc pattern formation [48] and imaginal disc-derived wing morphogenesis [49]. The insect wing is the most important organ for insect flight. Consequently, we chose, with regards to the RNAi for verification of gene function, *EGFR* as a representative that directly affects wing formation, thereby affecting flight capacity. Beside wings, flight muscles are also key organs that define the flight capacity [50]. The relevant genes directly determine the integrity of the structure and function of the flight muscles. Additionally, myofibrils are filled with mitochondria which regulate the energy supply to insect flight muscles, thereby regulating flight muscle formation and degradation [51]. The above transcriptomic analysis therefore suggests that *B. dorsalis* has advantages in wing vein morphogenesis, flight muscle structures and muscle energy supplement.

Flight consumes a large amount of energy that increases the metabolic rate from 50 to 100-fold above the usual state in the animal kingdom [52]. Common sources of energy for insect flight are lipids and carbohydrates, whose metabolism involves redox reactions. Lipid burning occurs in the mitochondria of the cell, and the mitochondrial matrix therefore provides an environment for lipid metabolism. It has been suggested that there is coordinated regulation of lipid metabolism based on the expression patterns of related enzymes [53,54]. The different unigenes expression levels for malate dehydrogenase, mitochondrial matrix and IPC revealed that *B. dorsalis* has a more active energy metabolism.

The effect of JH on flight has been described in some migratory insects to have a clear influence on flight and reproduction. Migratory activities begin when JH levels are low and stop when JH levels increase [55]. Our data support this conclusion. *JHBP* expression levels are higher in *B. correcta* that flies poorly, compared with *B. dorsalis*. Therefore, the down-regulation from *JHBP* is helpful for flight capacity.

We also found differential gene expression in the insulin signalling pathway. The insulin signalling pathway regulates the nutrients, energy metabolism, development and behaviour in insects [56,57,58]. For example, during wing formation and flight muscle development in alatae, there is a need for nutrient delivery and energy redistribution. Insulin receptors, which are the most upstream components of the insulin signalling pathway, play a key role in the transport of nutrients. Insulin signalling not only promotes glycogen synthesis and regulates fatty acid synthesis but also regulates JH synthesis, implying that insulin signalling is critical for regulating the energy supply and hormones in insect flight.

In the present study, it was evident that the disruption of *EGFR* influenced wing vein formation, thereby decreasing flight capacity. In view of the findings that *EGFR* knockdown led to severe effects on the athletic capacity and wing development, we suspected that *EGFR* is an important gene in overall larval to adult development as well as in the wing development, thus affecting the flight capacity of both species. The results of the diet-delivered RNAi in *B. dorsalis* and *B. correcta* in the present study suggested that oral administration of dsRNA efficiently suppressed target gene expression, and 1 µg of dsRNA was effective in knocking down the transcription of *EGFR* in an individual.

## 4. Materials and Methods

### 4.1. Insect Rearing

*B. dorsalis* and *B. correcta* were reared with an artificial diet at 25 ± 1 °C under a relative humidity of 70% and a 14 h:10 h L:D (Light:Dark) photoperiod. Papaya was used as a bait to lure the adults to lay eggs. Eggs and larvae were cultured using an artificial diet in glass bottles [59]. The third-instar larvae were transferred to wet soil for pupation. Adults emerged after approximately 10 days in the pupal stage and were placed in cages to complete their life cycle. *B. dorsalis* were collected from Guangzhou, Guangdong, China, and *B. correcta* were collected from Kunming, Yunnan, China. The populations were reared in the laboratory for approximately 20 generations.

### 4.2. Flight Capacity Tests

Experiments testing the flight capacity of *B. dorsalis* and *B. correcta* were conducted using a tethered flight mill system (Jiaduo Group, Hebi, Henan, China) [60,61,62]. Adults under slight ether anaesthesia were used in the tether experiments. The flight ring consisted of a twisted wire that fitted into a sleeve suspended on the flight arm, which was perpendicular to the axis. This setup allowed individuals to fly horizontally and rotationally. The pronotum of each insect was attached to the flight ring, and their head, thorax, belly and wings were not affected by the system. The experiments were conducted under a temperature maintained at 25 °C and a humidity of 40–70%. Each test was conducted from 9:00 a.m. to 9:00 p.m. under natural light. During flight, neither moisture nor the nutrition source was replenished. Unhealthy insects and those with damaged wings were not used in the experiments. After we ensured that the flight arm was balanced, the computer and flight mill system were engaged. The individual hanging on the flight mill flew along the tangent, and when one circular motion occurred, the control circuit of the flight mill produced one electrical signal. The computer then received a message to collect data from the system. The computer system automatically recorded flight parameters such as the average cumulative flight distance (km/head), the average cumulative flight time (h/head), the fastest speed (m/s), and the average speed (m/s). For each species, we tested and analyzed the flight capacity of 30 males and 30 females 12 days after eclosion.

### 4.3. RNA-Seq

The total RNA samples were isolated from adults of *B. dorsalis* and *B. correcta* (20 females and 20 males of each species) and from larvae (20 third-instar larvae) with TRIzol Reagent (Invitrogen, Carlsbad, CA, USA), following the manufacturer’s instructions. The total RNA was dissolved using RNase-free H_2_O. The quantity and integrity of the RNA were determined with a NanoDrop ND-2000 spectrophotometer (NanoDrop products, Wilmington, DE, USA) and an Agilent 2100 BioAnalyzer (Agilent Technologies, Englewood, CO, USA), following standard protocols. For use as a template, mRNA was enriched by removal of rRNA with a NEBNext Poly (A) mRNA Magnetic Isolation Module (NEB, E7490, Ipswich, MA, USA). A cDNA library was constructed using a NEBNext mRNA Library Prep Master Mix Set for Illumina (NEB, E6110, Ipswich, MA, USA) and NEBNext Multiplex Oligos for Illumina (NEB, E7500, Ipswich, MA, USA), according to the manufacturer’s recommendations. The enriched mRNA was randomly fragmented into 200–700 nt fragments by addition of an RNA fragmentation buffer. The mRNA fragments were reverse-transcribed into first-strand cDNA using random hexamers, followed by second-strand cDNA synthesis. The double-stranded cDNA was purified with a QiaQuick PCR extraction kit (Qiagen, Hilden, Germany), and cDNA products were processed via end repair, dA tailing and linkage of sequencing adaptors. The fragment sizes were assessed through 1.8% agarose gel electrophoresis. Finally, after validation by quantitative real-time PCR using a library quantification kit/Illumina GA Universal (KAPA, Wilmington, MA, USA), the library was sequenced on an Illumina HiSeq™ 2500 instrument (Illumina, San Diego, CA, USA) at the Biomarker Technologies company (Beijing, China). RNA-seq data are available at National Center for Biotechnology Information (NCBI): Sequence Read Archives SRP093863 and BioProject accession PRJNA353125.

### 4.4. Assembly and Annotation of the Transcriptome

Trinity, a transcriptomic de novo assembly program, was used to break down raw reads into short fragments (K-mers) [63], which were then extended into longer segments (contigs), and the overlap of contigs was used to obtain a fragment collection (component). Briefly, Trinity was used for assembly to obtain corresponding transcripts, and we selected the most important transcripts in the clustering unit as unigenes. The TGI Clustering Tool 2.1 was employed to assemble the transcripts into unigenes [64]. The unigenes were clustered again, and the longest were adopted to form non-redundant unigenes. For functional annotation, we obtained information on unigenes using BLAST with protein databases such as NR, Swiss-Prot, GO, COG, and KEGG [65,66,67,68,69,70]. From the NR annotations, we obtained GO annotations and KEGG pathways using the Blast2GO program [71]. All unigenes were searched against these databases using BLAST (ftp://ftp.ncbi.nlm.nih.gov/blast/executables/blast+/2.2.29/) (*e*-value < 10^−5^). Protein function was predicted according to the most similar proteins annotated in these databases. PCA was performed using the R language based on the prcomp function, which uses singular value decomposition, generally providing better numerical accuracy.

### 4.5. Differential Gene Expression Analysis

To detect DEGs between *B. dorsalis* and *B. correcta*, we used the empirical Bayes hierarchical model EBSeq [72]. In this analysis, we adopted a well-established Benjamini–Hochberg method to calibrate *p* values from the original assumption test [73]. After calibration, the *p* value was determined using the false discovery rate (FDR) to decrease false positives caused by an independent statistical hypothesis test of the expression of a large number of genes. During screening, we chose an FDR < 0.01 and a ∣fold-change (FC)∣ ≥ 2 as the criteria for a significant difference in expression between the two species. Hierarchical clustering analysis of DEGs was performed to cluster genes that exhibited the same or similar expression levels. DEGs were mapped to GO terms and KEGG pathways, and an enrichment analysis was performed to identify any over-representation of GO terms and KEGG pathways.

### 4.6. Synthesis of dsRNA and RNA Interference

We chose *EGFR* as a target gene to elucidate its function related to the flight capacity of *B. dorsalis* and *B. correcta.* On the basis of the transcriptomic data for the two species, gene-specific primers (ds*BdEGFR*-F/R; ds*BcEGFR*-F/R, Table S7) were designed to amplify a 399 bp fragment of *BdEGFR* and a 391 bp fragment of *BcEGFR.* GO Taq^®^ Hot Start DNA polymerase (Promega, Madison, WI, USA) was employed for amplification. The reverse transcription PCR (RT-PCR) product was subsequently gel recovered, purified and ligated into the pMD18-T vector (TAKARA, Tokyo, Japan). The vector was then transformed into *E. coli* DH5α competent cells (TAKARA) and sequenced. The ds*BdEGFR*, ds*BcEGFR*, ds*BdGFP* and ds*BcGFP* fragments were synthesized using the T7 RiboMAX™ Express RNAi System (Promega), according to the manufacturer’s protocol. The dsRNAs were dissolved in nuclease-free water (Promega), and their quality and concentration were determined via agarose gel electrophoresis and a Quawell UV-Vis Q5000 spectrophotometer (Quawell Technology Inc., San Jose, CA, USA) and were then stored at −20 °C until use.

Each first-instar larva of *B. dorsalis* or *B. correcta* was treated with approximately 1 µg of either ds*GFP* or ds*EGFR* mixed with 1.4 µL of the gene carrier G2 (1 mg/mL) [74]. The same amount of dsRNA was added 48 h later to achieve a continuous interference until pupation. Three biological replicates were performed for each treatment, and every biological replicate included 20 individuals. Mature larvae were picked and placed in the soil for pupation, and phenotypic comparisons were conducted after eclosion.

### 4.7. Quantitative Real-Time PCR (qRT-PCR)

The RNAi efficiency of ds*EGFR* was tested via qRT-PCR 48 h after the second feeding (primers in Table S7). Candidate genes were chosen for qRT-PCR analysis to verify the transcriptomic data. The total RNA of *B. dorsalis* and *B. correcta* in eight different developmental stages was extracted using the same method used for RNA-Seq. First-strand cDNA was synthesized from 1 μg of DNA-free RNA using the PrimeScript RT reagent kit with a gDNA Eraser (Perfect Real Time) (TAKARA). The cDNA products were used as templates, and details of the specific primers are listed in Table S8; 18S rRNA was used as an internal control. Next, qRT-PCR was performed with an Applied Biosystems 7500 Real-time PCR system (Life Technologies, Grand Island, NY, USA). The reaction mixture (25 μL total volume) contained 12.5 μL of SYBR^®^ Premix Ex Taq™ II (Tli RNaseH Plus) (TAKARA), 0.5 μL of ROX reference dye, 1 μL of each primer (10 μM), 1 μL of cDNA, and 9 μL of RNase-free water. The following thermal cycling profile was used: 95 °C for 30 s, 40 cycles of 95 °C for 5 s and 60 °C for 34 s, 95 °C for 15 s, 60 °C for 1 min and 95 °C for 15 s. Three independent biological replicates were performed. The relative expression was calculated via the 2^−ΔΔCt^ method [75].

### 4.8. Statistical Analysis

Student’s *t*-test was used for statistical data analysis. The R statistical programming language was applied for STL (Seasonal and Trend decomposition using Loess). The results of qRT-PCR are presented as the mean ± SD of three independent biological replicates. Comparisons between the means of two independent samples were performed with Student’s *t*-test, and multiple comparisons were performed with a one-way ANOVA and Duncan’s test in SPSS 17 (IBM Corporation, Armonk, NY, USA). A significant difference was considered to exist when *p* <0.05. Graphs were generated using SigmaPlot 13.0 (Systat Software Inc., San Jose, CA, USA).

## 5. Conclusions

The flight behavior of invasive insects is particularly important not only for their survival but also for expanding the scope of their life, then for colonization. The flight capacity is an important factor in this process. We focused on the differences in the flight capacity between *B. dorsalis* and *B. correcta* and identified candidate genes on a molecular level. It is a multi-factor regulation process involving a suite of underlying genes encoding enzymes including wing vein formation, energy metabolism, juvenile hormone, etc. RNAi indicated that *EGFR* plays a key role in the regulation of wing development and thereby in flight activity. These findings provided fundamental transcript information for exploring the flight capacity of insects and will facilitate our understanding of the molecular mechanisms of different flight performance in different insects.

## Supplementary Materials

Supplementary materials can be found at http://www.mdpi.com/1422-0067/19/2/396/s1.
