# Supplementary material for: Comparative Transcriptome Analyses Uncover Key Candidate Genes Mediating Flight Capacity in Bactrocera dorsalis (Hendel) and Bactrocera correcta (Bezzi) (Diptera: Tephritidae)
Source: Int J Mol Sci. 2018 Jan 30;19(2):396. doi: 10.3390/ijms19020396 (PMC5855618; doi:10.3390/ijms19020396)
Supplement: Supplementary file 1 [file ijms-19-00396-s001.pdf]

International Journal of
Molecular Sciences

MDPI

Supplementary Materials: Comparative Transcriptome Analyses Uncover Key Candidate Genes Mediating Flight Capacity in *Bactrocera dorsalis* (Hendel) and *Bactrocera correcta* (Bezzi) (Diptera: Tephritidae)

Shaokun Guo, Zihua Zhao, Lijun Liu, Jie Shen *, Zhihong Li *

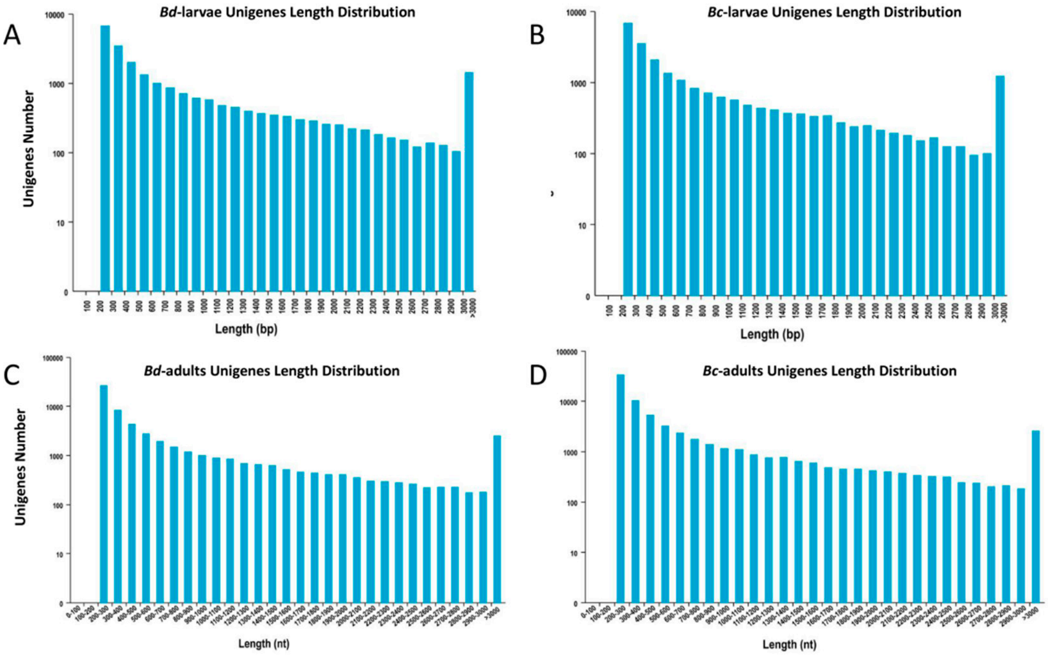

Figure S1. Unigenes length distribution of two species. Unigenes length distribution of *Bd*-larvae (A), *Bc*-larvae (B), *Bd*-adults (C), *Bc*-adults (D).

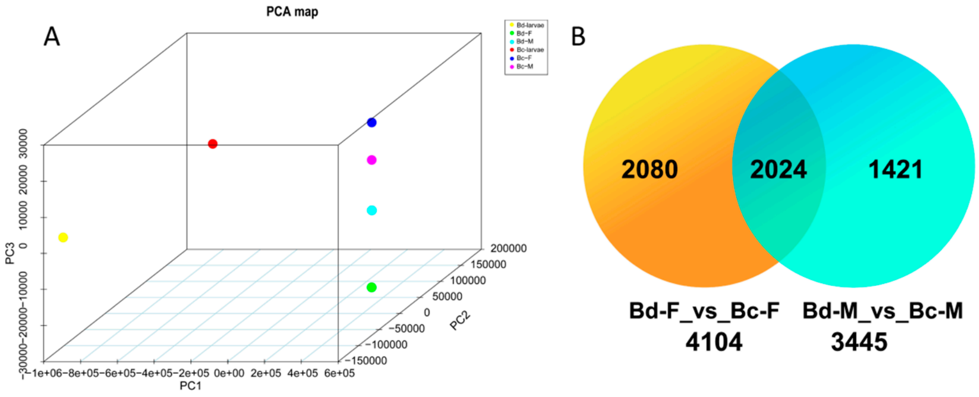

Figure S2. Essential information of transcriptome data. PCA plot of log_2-transformed read counts for each sample used in RNA-seq (A); Venn diagram showed the number of differential expressed genes between *Bd*-F vs *Bc*-F and *Bd*-M vs *Bc*-M (B).

Table S1. Detail information of flight mill experiments. The status of insects (age, gender), the fastest speed (km/h), the average speed(km/h), flight time sum (h), flight distance sum (km) were collected using the flight mill system.

ID	Insects name	Scientific name	Age (days)	Gender	Radius of flight arm(cm)	The fastest speed(km/h)	The average speed(km/h)	Flight time sum(h)	Flight distance sum(km)	Wind direction
1	oriental fruit fly	*B. dorsalis*	12	Female	15	3.39120	1.20236	0.567	0.68201	zero wind
2	oriental fruit fly	*B. dorsalis*	12	Female	15	2.71296	0.70627	0.336	0.23738	zero wind
3	oriental fruit fly	*B. dorsalis*	12	Female	15	4.74768	1.95823	0.369	0.72346	zero wind
4	oriental fruit fly	*B. dorsalis*	12	Female	15	4.06944	0.96656	3.149	3.0436	zero wind
5	oriental fruit fly	*B. dorsalis*	12	Female	15	3.39120	0.79793	0.293	0.23362	zero wind
6	oriental fruit fly	*B. dorsalis*	12	Female	15	4.06944	1.49753	0.530	0.79411	zero wind
7	oriental fruit fly	*B. dorsalis*	12	Female	15	3.39120	1.22471	1.418	1.73705	zero wind
8	oriental fruit fly	*B. dorsalis*	12	Female	15	4.74768	1.08275	0.310	0.33535	zero wind
9	oriental fruit fly	*B. dorsalis*	12	Female	15	2.71296	0.82800	0.916	0.75831	zero wind
10	oriental fruit fly	*B. dorsalis*	12	Female	15	4.06944	1.46738	3.104	4.55457	zero wind
11	oriental fruit fly	*B. dorsalis*	12	Female	15	3.39120	0.67022	1.057	0.70838	zero wind
12	oriental fruit fly	*B. dorsalis*	12	Female	15	4.06944	1.08410	2.021	2.19109	zero wind
13	oriental fruit fly	*B. dorsalis*	12	Female	15	4.06944	0.72613	0.366	0.26564	zero wind
14	oriental fruit fly	*B. dorsalis*	12	Female	15	2.71296	0.58491	0.868	0.50774	zero wind
15	oriental fruit fly	*B. dorsalis*	12	Female	15	4.06944	0.93860	0.231	0.21666	zero wind
16	oriental fruit fly	*B. dorsalis*	12	Female	15	2.03472	0.62521	0.288	0.17992	zero wind
17	oriental fruit fly	*B. dorsalis*	12	Female	15	3.39120	0.65256	0.359	0.23456	zero wind
18	oriental fruit fly	*B. dorsalis*	12	Female	15	2.71296	0.80209	0.160	0.12811	zero wind
19	oriental fruit fly	*B. dorsalis*	12	Female	15	3.39120	0.85238	0.566	0.4823	zero wind
20	oriental fruit fly	*B. dorsalis*	12	Female	15	2.03472	0.65376	0.362	0.23644	zero wind
21	oriental fruit fly	*B. dorsalis*	12	Female	15	3.39120	0.64278	0.085	0.05464	zero wind
22	oriental fruit fly	*B. dorsalis*	12	Female	15	4.06944	1.61171	1.836	2.95882	zero wind

23	oriental fruit fly	*B. dorsalis*	12	Female	15	10.17360	1.44292	0.695	1.00323	zero wind
24	oriental fruit fly	*B. dorsalis*	12	Female	15	3.39120	1.32437	0.188	0.24869	zero wind
25	oriental fruit fly	*B. dorsalis*	12	Female	15	4.06944	1.17044	0.706	0.82613	zero wind
26	oriental fruit fly	*B. dorsalis*	12	Female	15	9.49536	0.64982	1.213	0.78845	zero wind
27	oriental fruit fly	*B. dorsalis*	12	Female	15	3.39120	0.96469	0.478	0.46064	zero wind
28	oriental fruit fly	*B. dorsalis*	12	Female	15	2.03472	0.74820	0.097	0.07253	zero wind
29	oriental fruit fly	*B. dorsalis*	12	Female	15	2.71296	0.93181	0.580	0.54071	zero wind
30	oriental fruit fly	*B. dorsalis*	12	Female	15	9.49536	1.03480	3.025	3.13027	zero wind
31	oriental fruit fly	*B. dorsalis*	12	Male	15	7.46064	0.91414	1.157	1.05787	zero wind
32	oriental fruit fly	*B. dorsalis*	12	Male	15	2.71296	0.95149	0.193	0.18369	zero wind
33	oriental fruit fly	*B. dorsalis*	12	Male	15	3.39120	1.21838	0.493	0.60005	zero wind
34	oriental fruit fly	*B. dorsalis*	12	Male	15	2.03472	0.78036	0.196	0.1526	zero wind
35	oriental fruit fly	*B. dorsalis*	12	Male	15	2.03472	0.82200	0.274	0.22514	zero wind
36	oriental fruit fly	*B. dorsalis*	12	Male	15	5.42592	1.76940	3.089	5.46548	zero wind
37	oriental fruit fly	*B. dorsalis*	12	Male	15	4.06944	1.69965	0.233	0.39564	zero wind
38	oriental fruit fly	*B. dorsalis*	12	Male	15	3.39120	1.34345	0.231	0.31086	zero wind
39	oriental fruit fly	*B. dorsalis*	12	Male	15	3.39120	1.35472	1.286	1.7427	zero wind
40	oriental fruit fly	*B. dorsalis*	12	Male	15	2.71296	0.88223	0.219	0.19311	zero wind
41	oriental fruit fly	*B. dorsalis*	12	Male	15	2.03472	0.79393	0.249	0.19782	zero wind
42	oriental fruit fly	*B. dorsalis*	12	Male	15	3.39120	0.80156	0.367	0.2939	zero wind
43	oriental fruit fly	*B. dorsalis*	12	Male	15	2.03472	0.65956	0.333	0.21949	zero wind
44	oriental fruit fly	*B. dorsalis*	12	Male	15	2.71296	0.79571	0.375	0.29861	zero wind
45	oriental fruit fly	*B. dorsalis*	12	Male	15	4.74768	0.77126	0.243	0.18746	zero wind
46	oriental fruit fly	*B. dorsalis*	12	Male	15	3.39120	1.01002	0.167	0.16862	zero wind
47	oriental fruit fly	*B. dorsalis*	12	Male	15	2.71296	0.77058	0.284	0.21854	zero wind

48	oriental fruit fly	*B. dorsalis*	12	Male	15	2.71296	0.83139	1.148	0.95425	zero wind
49	oriental fruit fly	*B. dorsalis*	12	Male	15	3.39120	0.88964	1.300	1.15678	zero wind
50	oriental fruit fly	*B. dorsalis*	12	Male	15	2.71296	0.89755	0.223	0.1997	zero wind
51	oriental fruit fly	*B. dorsalis*	12	Male	15	3.39120	1.15442	0.693	0.79976	zero wind
52	oriental fruit fly	*B. dorsalis*	12	Male	15	6.78240	0.84276	0.748	0.6302	zero wind
53	oriental fruit fly	*B. dorsalis*	12	Male	15	4.74768	1.89879	3.333	6.3293	zero wind
54	oriental fruit fly	*B. dorsalis*	12	Male	15	3.39120	1.48266	2.376	3.52214	zero wind
55	oriental fruit fly	*B. dorsalis*	12	Male	15	2.71296	0.76342	0.234	0.17898	zero wind
56	oriental fruit fly	*B. dorsalis*	12	Male	15	2.71296	0.84058	0.620	0.52093	zero wind
57	oriental fruit fly	*B. dorsalis*	12	Male	15	5.42592	1.92634	1.851	3.56641	zero wind
58	oriental fruit fly	*B. dorsalis*	12	Male	15	2.71296	0.95224	0.307	0.29202	zero wind
59	oriental fruit fly	*B. dorsalis*	12	Male	15	3.39120	0.55333	0.344	0.19028	zero wind
60	oriental fruit fly	*B. dorsalis*	12	Male	15	4.06944	1.00617	1.524	1.53358	zero wind
61	guava fruit fly	*B. correcta*	12	Female	15	3.39120	1.21439	1.888	2.29283	zero wind
62	guava fruit fly	*B. correcta*	12	Female	15	3.39120	1.31999	0.176	0.23173	zero wind
63	guava fruit fly	*B. correcta*	12	Female	15	4.06944	0.81175	0.176	0.14318	zero wind
64	guava fruit fly	*B. correcta*	12	Female	15	10.17360	1.14799	1.910	2.19298	zero wind
65	guava fruit fly	*B. correcta*	12	Female	15	4.06944	1.34050	0.637	0.85345	zero wind
66	guava fruit fly	*B. correcta*	12	Female	15	2.71296	0.85776	1.183	1.01453	zero wind
67	guava fruit fly	*B. correcta*	12	Female	15	4.74768	0.99309	0.295	0.29296	zero wind
68	guava fruit fly	*B. correcta*	12	Female	15	3.39120	1.32030	0.349	0.46064	zero wind
69	guava fruit fly	*B. correcta*	12	Female	15	3.39120	0.70578	0.109	0.07724	zero wind
70	guava fruit fly	*B. correcta*	12	Female	15	3.39120	1.13493	0.969	1.10026	zero wind
71	guava fruit fly	*B. correcta*	12	Female	15	2.71296	0.74148	0.086	0.06406	zero wind
72	guava fruit fly	*B. correcta*	12	Female	15	3.39120	1.22687	0.299	0.36738	zero wind
73	guava fruit fly	*B. correcta*	12	Female	15	2.71296	0.62598	0.346	0.21666	zero wind
74	guava fruit fly	*B. correcta*	12	Female	15	6.10416	0.91759	0.388	0.35608	zero wind
75	guava fruit fly	*B. correcta*	12	Female	15	7.46064	0.69462	0.621	0.43144	zero wind
76	guava fruit fly	*B. correcta*	12	Female	15	2.71296	0.92778	0.059	0.05464	zero wind
77	guava fruit fly	*B. correcta*	12	Female	15	3.39120	1.01666	0.403	0.40977	zero wind
78	guava fruit fly	*B. correcta*	12	Female	15	2.71296	0.96621	0.050	0.04804	zero wind
79	guava fruit fly	*B. correcta*	12	Female	15	2.71296	0.73284	0.066	0.04804	zero wind
80	guava fruit fly	*B. correcta*	12	Female	15	8.81712	1.46810	0.466	0.68389	zero wind
81	guava fruit fly	*B. correcta*	12	Female	15	2.71296	0.80827	0.107	0.08666	zero wind
82	guava fruit fly	*B. correcta*	12	Female	15	4.06944	1.24529	0.085	0.1055	zero wind
83	guava fruit fly	*B. correcta*	12	Female	15	7.46064	1.07639	0.081	0.08761	zero wind

84	guava fruit fly	*B. correcta*	12	Female	15	4.74768	0.80827	0.161	0.13	zero wind
85	guava fruit fly	*B. correcta*	12	Female	15	3.39120	0.93051	0.046	0.04239	zero wind
86	guava fruit fly	*B. correcta*	12	Female	15	2.71296	1.12486	0.170	0.19123	zero wind
87	guava fruit fly	*B. correcta*	12	Female	15	2.71296	0.80395	0.064	0.05181	zero wind
88	guava fruit fly	*B. correcta*	12	Female	15	2.71296	0.68998	0.064	0.04427	zero wind
89	guava fruit fly	*B. correcta*	12	Female	15	4.06944	0.88125	0.697	0.61418	zero wind
90	guava fruit fly	*B. correcta*	12	Female	15	4.06944	0.91388	1.347	1.23119	zero wind
91	guava fruit fly	*B. correcta*	12	Male	15	2.03472	0.85657	0.322	0.27601	zero wind
92	guava fruit fly	*B. correcta*	12	Male	15	4.74768	1.24754	0.054	0.06688	zero wind
93	guava fruit fly	*B. correcta*	12	Male	15	3.39120	0.80890	0.581	0.47006	zero wind
94	guava fruit fly	*B. correcta*	12	Male	15	10.17360	1.77684	0.273	0.48419	zero wind
95	guava fruit fly	*B. correcta*	12	Male	15	4.74768	0.97625	0.073	0.07159	zero wind
96	guava fruit fly	*B. correcta*	12	Male	15	2.71296	1.16998	0.381	0.44557	zero wind
97	guava fruit fly	*B. correcta*	12	Male	15	3.39120	0.69530	0.221	0.15355	zero wind
98	guava fruit fly	*B. correcta*	12	Male	15	3.39120	0.94562	0.087	0.08195	zero wind
99	guava fruit fly	*B. correcta*	12	Male	15	3.39120	1.03432	0.056	0.05746	zero wind
100	guava fruit fly	*B. correcta*	12	Male	15	4.06944	0.86433	0.755	0.65281	zero wind
101	guava fruit fly	*B. correcta*	12	Male	15	3.39120	0.80263	0.271	0.2176	zero wind
102	guava fruit fly	*B. correcta*	12	Male	15	8.13888	0.82272	0.563	0.46346	zero wind
103	guava fruit fly	*B. correcta*	12	Male	15	2.71296	0.91817	0.281	0.25811	zero wind
104	guava fruit fly	*B. correcta*	12	Male	15	2.71296	0.85049	0.088	0.07442	zero wind
105	guava fruit fly	*B. correcta*	12	Male	15	3.39120	0.95913	0.055	0.05275	zero wind
106	guava fruit fly	*B. correcta*	12	Male	15	3.39120	0.92630	0.060	0.05558	zero wind
107	guava fruit fly	*B. correcta*	12	Male	15	2.71296	0.77198	0.068	0.05275	zero wind
108	guava fruit fly	*B. correcta*	12	Male	15	8.13888	1.57045	0.768	1.20576	zero wind
109	guava fruit fly	*B. correcta*	12	Male	15	10.17360	2.00179	0.549	1.09931	zero wind
110	guava fruit fly	*B. correcta*	12	Male	15	2.71296	0.87054	0.073	0.06311	zero wind
111	guava fruit fly	*B. correcta*	12	Male	15	4.06944	0.86615	0.064	0.05558	zero wind
112	guava fruit fly	*B. correcta*	12	Male	15	2.71296	0.95160	0.420	0.39941	zero wind
113	guava fruit fly	*B. correcta*	12	Male	15	4.06944	1.66599	0.604	1.007	zero wind
114	guava fruit fly	*B. correcta*	12	Male	15	3.39120	1.00543	0.055	0.05558	zero wind
115	guava fruit fly	*B. correcta*	12	Male	15	2.71296	0.86992	0.064	0.05558	zero wind
116	guava fruit fly	*B. correcta*	12	Male	15	2.71296	0.80494	0.901	0.72534	zero wind
117	guava fruit fly	*B. correcta*	12	Male	15	3.39120	0.77698	0.116	0.09043	zero wind
118	guava fruit fly	*B. correcta*	12	Male	15	3.39120	0.89270	0.178	0.1592	zero wind
119	guava fruit fly	*B. correcta*	12	Male	15	2.71296	0.70899	0.110	0.07819	zero wind
120	guava fruit fly	*B. correcta*	12	Male	15	3.39120	1.02108	0.405	0.41354	zero wind

Table S2. Statistics for larvae unigenes assessment.

Length range	*Bd*-larvae	*Bc*-larvae	All Unigenes
200-300	6625(28.34%)	6810(28.90%)	10155(29.67%)
300-500	5433(23.24%)	5577(23.67%)	8007(23.39%)
500-1000	4479(19.16%)	4576(19.42%)	6395(18.68%)
1000-2000	3771(16.13%)	3791(16.09%)	5168(15.10%)
2000+	3071(13.14%)	2809(11.92%)	4505(13.16%)
Total number	23379	23563	34230
Total length	22708737	21847818	33189030
N50 length	1822	1705	1875
Mean length	971.3305531	927.2086746	969.5889571

Table S3. Statistics for adults unigenes assessment.

Length range	*Bd*-adults Unigenes	*Bc*-adults Unigenes	All Unigenes
200-300	26,461(45.31%)	33,421(47.21%)	52,503(47.35%)
300-500	12,718(21.78%)	15,651(22.11%)	24,094(21.73%)
500-1000	8,321(14.25%)	9,844(13.91%)	15,487(13.97%)
1000-2000	5,909(10.12%)	6,499(9.18%)	9,898(8.93%)
2000+	4,990(8.54%)	5,377(7.60%)	8,908(8.03%)
Total Number	58,399	70,793	110,890
Total Length	42,860,128	48,570,878	78,529,526
N50 Length	1,505	1,353	1,456
Mean Length	733.92	686.1	708.17

Table S4. A total of 19829 unigenes were annotated in larvae transcriptome from NR, Swiss-Prot, GO, COG and KEGG databases.

Anno_Database	Annotated_Number	300<=length<1000	length>=1000
COG_Annotation	5189	1262	3639
GO_Annotation	13461	4254	7563
KEGG_Annotation	5420	1518	3411
Swissprot_Annotation	12790	3969	7587
nr_Annotation	19786	7295	9241
All_Annotated	19829	7318	9244

Table S5. A total of 26368 unigenes were annotated in *Bd*-adults transcriptome from NR, Swiss-Prot, GO, COG, KOG, KEGG and Pfam.

Anno_Database	Annotated_Number	300<=length<1000	length>=1000
COG_Annotation	8929	2643	3384
GO_Annotation	13432	3982	6646
KEGG_Annotation	8577	2544	4325
KOG_Annotation	14770	4479	6820
Pfam_Annotation	16313	5040	7512
Swissprot_Annotation	11954	3629	6026
nr_Annotation	23962	8318	9505
All_Annotated	26368	9080	9558

Table S6. A total of 27939 unigenes were annotated in *Bc*-adults transcriptome from NR, Swiss-Prot, GO, COG, KOG, KEGG and Pfam.

Anno_Database	Annotated_Number	300<=length<1000	length>=1000
COG_Annotation	9448	2705	3375
GO_Annotation	13178	3868	6800
KEGG_Annotation	8397	2493	4357
KOG_Annotation	14874	4382	6932
Pfam_Annotation	16989	5193	7687
nr_Annotation	25039	8677	9809
All_Annotated	27939	9512	9873

Table S7. Primers for dsRNA synthesis and RNAi efficiency test.

Name	Primer	5′-3′	Length	Target fragment
ds*BdEGFR*	F	GACTGGTGCATCGTGATCTG	20	399
	R	CCAGCTGTTTGAATGATGGA	20	
ds*BcEGFR*	F	CTGAGGGAGGCATATATCATGG	22	391
	R	CACGATGTCTGATGCACTCC	20	
BdEGFR-qPCR	F	CGGCTACATACTCATCAGTCA	21	138
	R	AACATTTTCGAGTAGGCAGT	20	
BcEGFR-qPCR	F	TTACACTCGGGCAACATTGA	20	123
	R	GCGTGGACCAAACGAGTTAT	20	

Table S8. Primers for qRT-PCR verification of 7 genes.

Gene name	Primer	5′-3′	Length	Target fragment
Malate	F	GCAGAATAGAGCCTTTGGACA	21	131
dehydrogenase	R	TTGTGATGGTGGTAGGTGGT	20	
Mitochondrial	F	GGAACGTATGGTGGAGGGTA	20	119
matrix	R	CGTACTTCGGCAATAGCACA	20	
Inorganic	F	GTAAGCGTACATGCCGTTCA	20	105
phosphate cotransporter	R	TCAGTGCCCTATTTGGTGCT	20	
Diptericin	F	CCCAAAGACAGCCTCAGTTC	20	157
	R	TATCGTCCGCCCAAATGT	18	
Tachykinin-like	F	TCCATTGTGCTACGTTTCCA	20	121
peptides receptor 86C	R	GTTGTTGCTATGGTGCAGTCA	21	
Serine protease	F	TCCGCGAAAGTTTTCTGTTT	20	167
	R	CAAGCAACAACTGGCAGCTA	20	
EGFR	F	CGGCTACATACTCATCAGTCA	21	138
	R	AACATTTTCGAGTAGGCAGT	20	
